# The alternative oxidase (AOX) increases sulphide tolerance in the highly invasive marine invertebrate *Ciona intestinalis*

**DOI:** 10.1242/jeb.242985

**Published:** 2021-08-26

**Authors:** Katharina Bremer, Hitoyoshi Yasuo, Paul Vincent Debes, Howard Trevor Jacobs

**Affiliations:** 1 Tampere University, Faculty of Medicine and Health Technology, 33014 Tampere, Finland; 2 Laboratoire de Biologie du Développement de Villefranche-sur-mer, Institut de la Mer de Villefranche, Sorbonne Université, CNRS, 06230 Villefranche-sur-mer, France; 3 Hólar University College, Department of Aquaculture and Fish Biology, 551 Sauðárkrókur, Iceland

**Keywords:** Climate change, Embryonic development, Energy metabolism, Invasive species, Morpholino

## Abstract

Ecological communities and biodiversity are shaped by both abiotic and biotic factors. This is well illustrated by extreme environments and invasive species. Besides naturally occurring sulphide-rich environments, global change can lead to an increase in hydrogen sulphide episodes that threaten many multicellular organisms. With the increase in the formation, size and abundance of oxygen minimum zones and hypoxic environments, bacterial-associated sulphide production is favoured and, as such, hydrogen-sulphide-rich environments are likely to also increase in size and abundance. Many species are challenged by the inhibiting effect of sulphide on aerobic energy production via cytochrome *c* oxidase, ultimately causing the death of the organism. Interestingly, many protist, yeast, plant and also animal species possess a sulphide-resistant alternative oxidase (AOX). In this study, we investigated whether AOX is functionally involved in the sulphide stress response of the highly invasive marine tunicate *Ciona intestinalis*. At the LC_50_, the sulphide-induced reduction of developmental success was three times stronger in *AOX* knock-down embryos than in control embryos. Further, *AOX* mRNA levels were higher under sulphide than under control conditions, and this effect increased during embryonic development. Together, we found that AOX is indeed functionally involved in the sulphide tolerance of *C. intestinalis* embryos, hence, very likely contributing to its invasive potential; and that the response of AOX to sulphide seems to be controlled at the transcriptional level. We suggest that AOX-possessing species play an important role in shaping marine ecological communities, and this importance may increase under ongoing global change.

## Abstract (precis)

**Summary:** A functional study on the role of the mitochondrial alternative oxidase (AOX) in an animal species indicates that AOX increases the sulphide tolerance of the marine chordate *Ciona intestinalis*.

## INTRODUCTION

Ecological communities and biodiversity are shaped by two main factors, climate change and invasive species (Mainka and Howard, 2010). With the currently ongoing change in climate, many environments have already experienced alterations in their characteristics, and will continue to do so, while extremes increase in abundance and severity, consequently affecting ecological communities (Bellard et al., 2012; Mainka and Howard, 2010; Worm and Lotze, 2021). Invasive species play another important role in shaping ecological communities and biodiversity (Bax et al., 2003; Molnar et al., 2008). By nature, invasive species have to be well prepared to cope with diverse stressors, and this in turn may be one key feature that enables them to compete in a new environment. Therefore, understanding the underlying mechanisms of how organisms in general and invasive species in particular respond to extreme environments is crucial to improve predictions for ecological communities and biodiversity, especially in the context of climate change.

The oceans, covering approximately 70% of the Earth's surface, play a central role in climate change. With increased carbon dioxide levels in the atmosphere, the oceans are greatly affected by changing temperature (becoming warmer) and pH (becoming more acidic). The consequences of those changes are manifold, ranging from less oxygenated oceans to rises in sea levels, changes in ocean currents and increasing weather extremes (Diaz and Rosenberg, 2008). One additional consequence with considerable impact on the survival of multicellular organisms has rarely been mentioned in the climate change debate: the increase in hydrogen sulphide (H_2_S) in the oceans. In general, high sulphide concentrations are due to geothermal and biological processes (Bagarinao, 1992). Interestingly, biological processes relate to oxygen levels: with depleted oxygen levels, bacterial sulphate reduction takes place and H_2_S is produced (Jørgensen et al., 1982; Schunck et al., 2013), and eventually moves up the water column (Bakun and Weeks, 2004; Brüchert et al., 2006; Stewart, 2011). This sulphide–oxygen link becomes rather important because the formation, size and abundance of oxygen minimum zones and anoxic bottom waters are increasing (Breitburg et al., 2018; Diaz and Rosenberg, 2008; Stramma et al., 2010), consequently causing bacterial-associated sulphide production.

The reason for the detrimental potential of H_2_S is the inhibition of the aerobic energy production pathway (Bouillaud and Blachier, 2011; Cooper and Brown, 2008). Most eukaryotic organisms produce their energy in the form of adenosine triphosphate (ATP), the majority of which is produced in mitochondria via oxidative phosphorylation (OXPHOS). In this pathway, H_2_S inhibits cytochrome *c* oxidase (COX), which limits or blocks ATP production, ultimately leading to the death of the organism. However, diverse ecological communities are found around in environments with high H_2_S concentrations that are lethal for most species, including deep-sea hydrothermal vents, cold seeps, marine sediments, freshwater springs and caves (Riesch et al., 2015; Tobler et al., 2016). Those species have manifold behavioural, physiological and biochemical mechanisms to avoid or eliminate H_2_S (Abel et al., 1987; Greenway et al., 2014; Jahn et al., 1997; Laudien et al., 2002; Miron and Kristensen, 1993; Pfenninger et al., 2014). Interestingly, many species among almost all kingdoms of life possess a sulphide-resistant, non-protonmotive alternative oxidase (AOX). AOX can substitute for COX and thus ensures the maintenance of energy production, albeit at a decreased level, as well as preserving mitochondrial redox homeostasis, both of which are crucial for mitochondrial function and, ultimately, survival. In protists, fungi, plants and also some animal species, AOX has been shown to be involved in coping with a multitude of abiotic and biotic stressors impairing OXPHOS, such as extreme temperatures, nutrient limitations, metals and pathogen infections (McDonald, 2008; Rogov et al., 2014; Saari et al., 2019; Tward et al., 2019).

In this context, the tunicate *Ciona intestinalis* is of great interest for two reasons. First, *C. intestinalis* is a highly successful invasive marine invertebrate that imposes considerable ecological impacts on newly invaded communities (Blum et al., 2007; Therriault and Herborg, 2008). Second, *C. intestinalis* possesses AOX (McDonald and Vanlerberghe, 2004), which may be part of the reason for its tolerance to sulphide and, hence, its invasive potential.

In adult *C. intestinalis*, elevated H_2_S concentrations elicit a strong response in the *AOX* transcript levels in heart and neural complex tissue (Saari et al., 2019), indicating that AOX may play a role in mitigating H_2_S stress in adults. However, a functional verification of the transcript level response is still lacking and the effect of H_2_S on the early developmental stages of *C. intestinalis* remains unknown. This information, however, is of great interest. These early developmental stages are those that invade new habitats and ecological communities. However, early developmental stages have also been shown to be more susceptible to external stressors compared with adult stages in many species (Pineda et al., 2012).

Although *AOX* has been detected at the sequence and transcript levels in many animal phyla from Placozoa to Chordata (McDonald et al., 2009; Tward et al., 2019), studies on the functional role of AOX will improve our understanding of how animals tolerate and invade potentially harsh environments. Hence, our aim was to understand the role of AOX during early development of the highly invasive ascidian *C. intestinalis*. Specifically, we tested whether a lack of AOX protein during early *C. intestinalis* development causes lower developmental success under elevated H_2_S concentrations, relative to control groups. With this study, we aimed to shed light on the ecological implication of AOX in *C. intestinalis* and consequently other AOX-possessing animals, and thereby encourage a new perspective on the alternative mitochondrial pathway in the context of the ecology of aquatic communities and climate change.

## MATERIALS AND METHODS

We conducted all animal experiments and survival analyses at the Observatoire Océanologique de Villefranche-sur-Mer, France. The mRNA analyses were performed at the BioMediTech, Tampere University, Finland.

### Animals

Adult *Ciona intestinalis* (Linnaeus 1767) were collected off the coast of Brest, France, by the Centre de Ressources Biologiques Marines of the Station Biologique de Roscoff. Prior to experiments, animals were kept in an 18°C, temperature-controlled flow-through system with natural seawater at the station. A detailed visualisation and description of *C. intestinalis* embryonic development is provided by Hotta et al. (2007).

### Experimental set-up and sampling procedure

We performed all experiments at 18°C using artificial seawater (ASW, 420 mmol l^−1^ NaCl, 9 mmol l^−1^ KCl, 10 mmol l^−1^ CaCl_2_, 24.5 mmol l^−1^ MgCl_2_, 25.5 mmol l^−1^ MgSO_4_, 2.15 mmol l^−1^ NaHCO_3_, 10 mmol l^−1^ Hepes buffer, pH 8.0, 0.05 g l^−1^ kanamycin sulphate to prevent potential bacterial growth in embryo cultures, sterilized with a 0.22 µm filter) and 1% agarose-coated Petri dishes (Ø 55 mm, Gosselin, Hazebrouck, France) (Sardet et al., 2011). In all experiments, we used unrelated families as biological replicates, i.e. sperm and eggs for each family were only used once. On the day of the experiment, eggs and sperm for each family were freshly sampled and eggs were dechorionated and fertilized according to Sardet et al. (2011). For all sulphide treatments, we used a sodium sulphide (Na_2_S, 99.99% trace metals basis, no. 431648, Sigma Aldrich, St Louis, MO, USA) stock solution (10 mmol l^−1^) and used specific volumes to obtain the desired final concentration. In solution, Na_2_S dissociates into three sulphidic species that equilibrate quickly into sulphide (S^2−^), sulfanide (HS^−^) and hydrogen sulphide (H_2_S). At the average physiological pH of 7.4, H_2_S is present at approximately 20–30% and HS^−^ at 70–80%, while S^2−^ is only present in negligible quantity (Szabo et al., 2014). For simplicity and comparable reasons with previous studies that use this approach, we hereafter use ‘sulphide’ to refer to all three species collectively.

#### Sulphide dose–response experiment

To determine the effect of sulphide on the developmental success of *C. intestinalis* and the concentration that resulted in 50% embryos being dead before completing development to tailbud stage (LC_50_), we tested five different sulphide concentrations (0, 10, 20, 25 and 50 μmol l^−1^) on five families with technical duplicates (two Petri dishes per family) per concentration for three of the five families. After fertilization, we distributed zygotes of each family evenly across treatment dishes filled with 10 ml of ASW and directly added sulphide at concentration-specific amounts. We terminated the experiment after 18.5 h of post-fertilization development by adding paraformaldehyde (PFA) to each dish. We then analysed the successful development to tailbud stage by counting the number of embryos that did and did not fully develop to that stage using a stereo microscope and calculating the proportion of those embryos that fully developed to tailbud stage.

#### AOX knock-down experiment

**Fig. 1.**
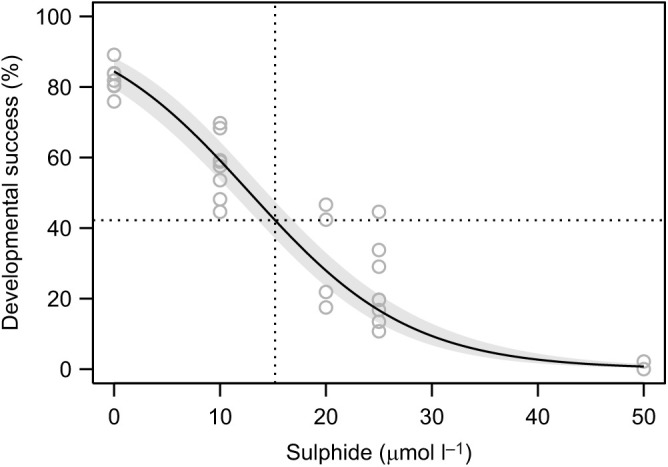
**Developmental success of *Ciona intestinalis* embryos in response to sulphide.** Back-transformed model prediction for the regression curve with 95% confidence band; light grey circles show family means. The 50% observed developmental success relative to the experimental maximum (LC_50_) was determined to be at a sulphide concentration of 15.2 μmol l^−1^ according to the estimated model coefficients (±s.e.): logit developmental success=1.689(±0.163)–0.132(±0.009)×[sulphide].

In order to investigate whether AOX plays a role in the tolerance to sulphide in *C. intestinalis* embryos, we conducted knock-down experiments using a morpholino (MO) anti-sense oligomer targeted to the 5'-untranslated region (UTR) of *AOX* transcripts to block their translation. The set-up for the MO injections is described in Yasuo and McDougall (2018). We sampled eggs and sperm following the same protocol as in the dose–response experiment and tested seven *C. intestinalis* families on three ‘MO groups’ and four of those families in a rescue group: (1) uninjected eggs (uninjected), (2) control-MO injected eggs (MO_Ctrl_), (3) AOX-MO injected eggs (MO_AOX_) and (4) AOX-MO and MO-immune *AOX* mRNA-injected eggs (MO_AOX_+*AOX* mRNA). The rescue group addresses the specificity of AOX-MO; the MO-immune *AOX* mRNA consists of the *AOX* open reading frame [obtained from pWPI-AOX (HindIII); courtesy of Dr Eric Dufour, Faculty of Medicine and Health Technology, Tampere University, Finland] and re-cloned into the pBluescript RN3 vector (Lemaire et al., 1995). The standard control-MO (CCTCTTACCTCAGTTACAATTTATA) (Gene Tools, LLC) and AOX-MO (TTCCGGTAGACAACATATTTGTTGC) (Gene Tools, LLC) were injected at 1 mmol l^−1^, while *AOX* mRNA was injected at 0.5 μg μl^−1^. The control-MO targets a human β-globin intron mutation that causes beta-thalassemia and is broadly used as a negative control and it causes no visible phenotype in *C. intestinalis*. *AOX* mRNA was synthesized from linearized pRN3-AOX-3HA using the mMESSAGE mMACHINE T3 Kit (Ambion, Thermo Fisher, AM1348). Approximately 1 h after injection and no injection for the uninjected control group, eggs were fertilized and selected for correctly developed embryos when they reached the 8-cell stage. We then distributed embryos of each MO group equally into two Petri dishes (∼50 eggs per dish) and started the experiment by adding sulphide to one of the two dishes to obtain a final concentration of 15 μmol l^−1^ in a final volume of 10 ml ASW (LC_50_; Fig. 1). As with the dose–response experiment, we terminated this experiment after 18.5 h of development by adding PFA to each dish and determined the proportion of embryos showing successful development to tailbud stage.

#### AOX transcript levels during *C. intestinalis* development under sulphide stress

To test the effect of sulphide on *AOX* transcript levels along developmental stages, we exposed developing *C. intestinalis* embryos to either LC_50_ sulphide (15 μmol l^−1^) or control (no sulphide) conditions. Embryos of four families were collected at egg (control condition only), 32-cell, mid-gastrula, mid-neurula and mid-tailbud stages and immediately frozen and stored at −80°C. We extracted RNA (Arcturus PicoPure RNA Isolation Kit, ABI, Foster City, CA, USA) and removed residual genomic DNA by treatment with RNase-free DNase I (Thermo Fisher Scientific, Waltham, MA, USA) according to the manufacturer's protocol, followed by reverse transcription (High-Capacity cDNA Reverse Transcription Kit, ABI, Foster City, CA). Extracted RNAs were quantified spectrophotometrically (NanoDrop 2000, Thermo Fisher Scientific). We determined transcript levels based on qPCR (ABI StepOne Plus instrument and Fast SYBR Green Master Mix, ABI) with gene-specific primer sets [*AOX*: efficiency=1.81, (Saari et al., 2019); *β-actin*: efficiency=1.90, (Fujikawa et al., 2010); *TCEB3* (transcription elongation factor B polypeptide 3): efficiency=1.87, forward primer (5′–3′) GTATGCCTGGTACGATTCCCAACT, reverse primer (5′–3′) AAACTGCCTATCTTCCAAAGATGCTC (present study)]. On each of four 96-well plates, we included sample duplicates for *AOX* and two housekeeping genes (*β-actin*, *TCEB3*) and no-template controls per gene (no contaminations detected). We used the online tool Real-time PCR Miner (Zhao and Fernald, 2005) to estimate quantification cycle (*C*_q_) values and amplification efficiencies for each qPCR replicate (*E*; subsequently averaged per gene and plate) from the raw qPCR fluorescence data. To allow analysis of *C*_q_ values via linear models, we efficiency-standardized *C*_q_ values as *E*^*C*_q_^ per gene and plate and then standardized to a common log_2_ *C*_q_ scale across genes and plates as log_2_(*E*^*C*_q_^). Accordingly, all linear model contrasts represent the log_2_ of traditionally used *C*_q_ ratios.

### Statistical analyses

We analysed the dose–response data using a generalized linear mixed model with logit-link function and binomial error distribution under Laplace approximation to the likelihood as implemented in the R function glmer of the R package lme4 (Bates et al., 2015). The proportions of individuals that survived were modelled with a fixed covariate for sulphide concentration (in μmol l^−1^; representing a logit regression), and random terms for family, family×concentration and family×concentration×replicate (accounting for detected overdispersion). We determined the LC_50_ relative to the survival at sulphide absence based on the estimated model intercept=1.689±0.163 (estimate±s.e.) and regression slope=−0.132±0.009 as 15.2 μmol l^−1^ sulphide, which we rounded to 15 μmol l^−1^ for use in the *AOX* knock-down experiment.

We analysed the *AOX* knock-down experiment data using a generalized linear mixed model with logit link function like for the dose–response data. The developmental success binaries (embryos that developed to tailbud stage relative to all embryos) were modelled with fixed terms for the sulphide treatment (15 μmol l^−1^ or no sulphide), the MO groups and their interaction, and random terms for family, family×treatment and family×MO. We performed pairwise comparisons of predicted means for each MO group within each treatment and between treatments within each MO group using *t*-tests and adjusted the *P*-values for the false discovery rate (Benjamini and Hochberg, 1995).

We analysed the qPCR data using linear mixed models. We first fitted a model to test for a constant log_2_ *C*_q_ across development for each of the two housekeeping genes and a constant difference between them, which held for the latter but not the former. Next, we averaged the *C*_q_ across housekeeping genes per family, treatment and developmental stage. We then standardized *AOX C*_q_ values by subtracting the average housekeeping *C*_q_ values. These efficiency- and housekeeping-gene-standardized AOX *C*_q_ values were modelled with fixed terms for the sulphide treatment (treatment: 15 μmol l^−1^, no sulphide), the five stages sampled [stage: egg (control condition only), 32-cell, gastrula, neurula, tailbud] and their interaction, and random terms for family, family×treatment, family×stage and family×stage×treatment. We tested fixed terms using *F-*tests. We performed multiple comparisons of treatment contrasts within each stage similarly as reported for the *AOX* knock-down experiment.

## RESULTS

In our study, we used an MO-based knock-down approach to test whether AOX plays a role in successful embryonic development of *C. intestinalis* under elevated sulphide concentrations. First, we determined the sulphide concentration at which 50% of developmental success to tailbud stage was compromised (sulphide LC_50_). Then, we tested whether *AOX* knock-down affected developmental success at those concentrations. Lastly, we gained insight into *AOX* mRNA responses during development in the presence and absence of LC_50_ sulphide concentrations.

### Elevated hydrogen sulphide levels reduce *C. intestinalis* developmental success

The dose–response experiment indicated that *C. intestinalis* larval development success depended strongly on the sulphide concentration (Fig. 1). The average maximum developmental success in the absence of sulphide was 84%, whereas at sulphide concentrations around 50 µmol l^−1^, absolute developmental success was approaching zero. Based on the logistic mixed model, we determined a sulphide LC_50_ of ∼15 µmol l^−1^ compared with embryos not exposed to sulphide. We used this concentration in the following experiments.

### Knock-down of *AOX* reduces the tolerance to sulphide

**Fig. 2.**
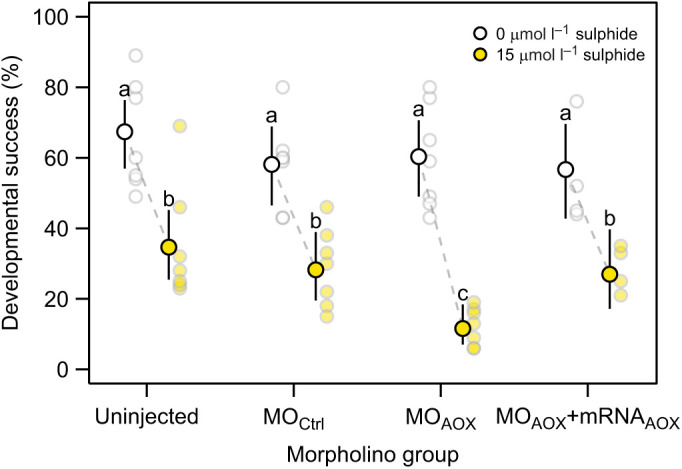
**Developmental success of *C. intestinalis* embryos of different morpholino (MO) groups (uninjected, MO_Ctrl_, MO_AOX_, MO_AOX_+mRNA_AOX_) under either LC_50_ sulphide or absence of sulphide.** Back-transformed model predictions for sulphide treatment×MO group means±95% CI; light grey circles show counted family means. We ran seven biological replicates (families) for all MO groups per treatment except for the rescue group, for which we ran four families. Different letters indicate groups with significant difference at FDR <0.05.

In the absence of sulphide, we did not detect any significant difference in developmental success among the four MO groups (Fig. 2, Table 1), indicating that injection procedures had little impact on developmental success. In contrast, when embryos were exposed to sulphide LC_50_, the developmental success decreased within each of the four MO groups compared with control conditions without sulphide (Fig. 2, Table 2).

**Table 1.**
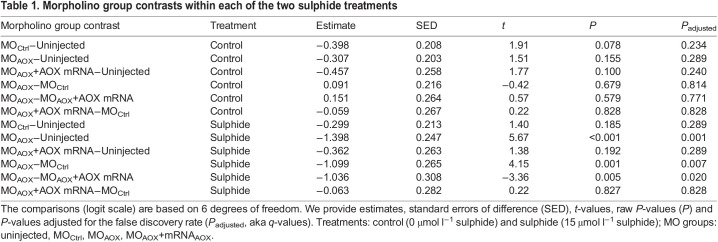
Morpholino group contrasts within each of the two sulphide treatments

**Table 2.**
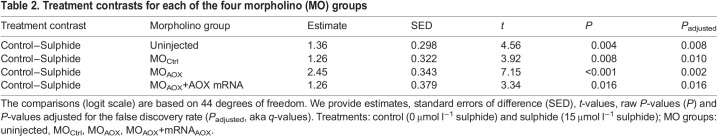
Treatment contrasts for each of the four morpholino (MO) groups

The developmental success under sulphide LC_50_ decreased for the uninjected embryos by 49% [from 67% (95% CI 57–76%) to 35% (95% CI 26–45%)], for the MO_Ctrl_-injected by 51% [from 58% (95% CI 47–69%) to 28% (95% CI 20–39%)] and for the rescue group by 52% [from 57% (95% CI 43–70%) to 27% (95% CI 17–40%)]. Importantly, however, the AOX-MO group exhibited a stronger effect of the sulphide LC_50_ than any of the other MO groups; the developmental success decreased by 81% [from 60% (95% CI 49–71%) to 12% (95% CI 7–18%)] (Fig. 2). These sulphide effects can be translated into odds ratios, i.e. the odds for an embryo to survive in control conditions relative to sulphidic conditions within each MO group. For the uninjected group, the odds ratio was 3.9 (95% CI 2.1–7.1), for the MO_Ctrl_ group, it was 3.5 (95% CI 1.9–6.7), and for the rescue group, it was 3.5 (95% CI 1.7–7.6). For the *AOX* knock-down group, the odds to survive under control conditions were 11.6 (95% CI 5.9–23.0) times higher than to survive under sulphidic conditions. Further, to evaluate the relative effect of AOX, we contrasted the odds ratio of the *AOX* knock-down group with that of each of the two control MO groups. In other words, we quantified the ratio of developmental success of control embryos (uninjected and MO_Ctrl_-injected) and *AOX* knock-down embryos under sulphide conditions to control conditions. We found the odds to survive under LC_50_ sulphide conditions were higher for control embryos than for *AOX* knock-down embryos. Specifically, we found the odds to survive under LC_50_ sulphide conditions for the uninjected control group to be three times higher (95% CI 1.7–5.3) and for the MO_Ctrl_ group 3.3 times higher (95% CI 1.8–6.2) compared with the *AOX* knock-down group.

### AOX transcript levels at different developmental stages under sulphide exposure

We detected differences in the effects of developmental stage on housekeeping gene transcript levels (gene×stage; Table 3), i.e. the housekeeping gene transcript levels change with development asynchronously, which hinders an interpretation of the results as changes across development. However, the developmental stage effect on transcript levels between housekeeping genes did not differ between the 15 and 0 μmol l^−1^ sulphide treatments (gene×stage×treatment; Table 3). This absence of stage×treatment interaction for the housekeeping genes (used for the *AOX C*_q_ standardization) allows for valid AOX transcript level contrasts between sulphide treatments. Therefore, we performed treatment contrasts, i.e. the difference in *AOX* mRNA levels (relative to housekeeping gene levels) between sulphide and control treatments for each developmental stage, but we did not perform treatment contrasts across development.

**Table 3.**
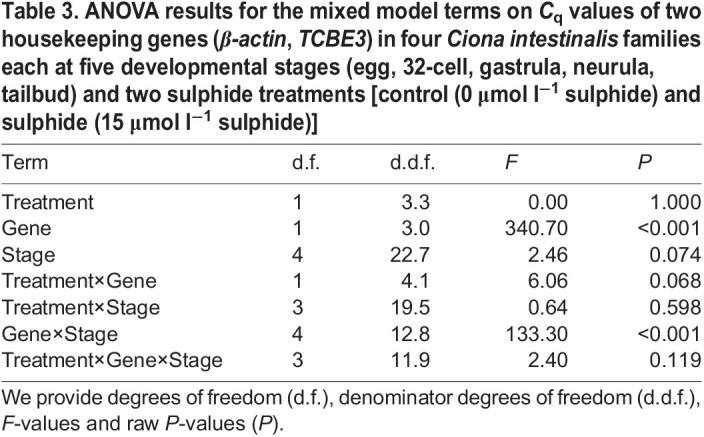
ANOVA results for the mixed model terms on *C*_q_ values of two housekeeping genes (*β-actin*, *TCBE3*) in four *Ciona intestinalis* families each at five developmental stages (egg, 32-cell, gastrula, neurula, tailbud) and two sulphide treatments [control (0 μmol l^−1^ sulphide) and sulphide (15 μmol l^−1^ sulphide)]

**Fig. 3.**
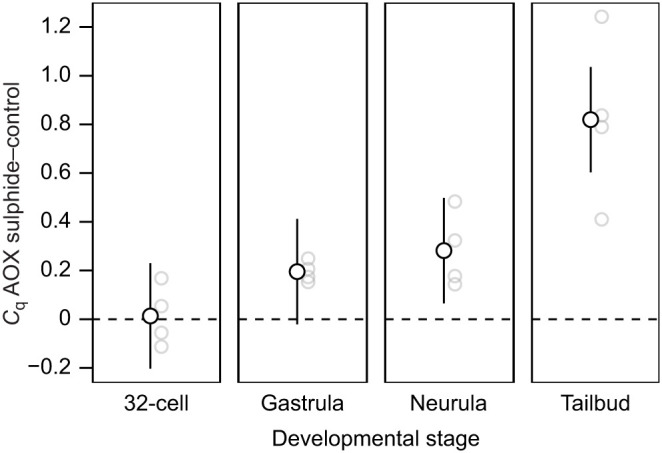
**Differences in *AOX* transcript levels between LC_50_ sulphide (15 μmol l^−1^ sulphide) and control (0 μmol l^−1^ sulphide) treatments at four different developmental stages.** Back-transformed model predictions for the mean±95% CI treatment contrasts (*C*_q_ AOX sulphide–control) per developmental stage; light grey circles show model predicted contrasts for each family (*n*=4). *P*-values are based on *t*-tests with 12.1 degrees of freedom and are adjusted for the false discovery rate (*N*=4; 32-cell: *t*=0.12, *P*=0.905, gastrula: *t*=1.80, *P*=0.128, neurula: *t*=2.60, *P*=0.046, tailbud: *t*=7.57, *P*=<0.001).

Relative *AOX* transcript levels between the 15 and 0 µmol l^−1^ sulphide-exposed embryos were very similar at the 32-cell stage, while their difference increased significantly at the neurula and tailbud stages (Fig. 3). Further, the *AOX* mRNA level differences between the control and sulphide treatment for the 32-cell, gastrula and neurula stages were similar, while the *AOX* mRNA level difference was significantly higher for the tailbud stage (Tables 4 and 5).

**Table 4.**
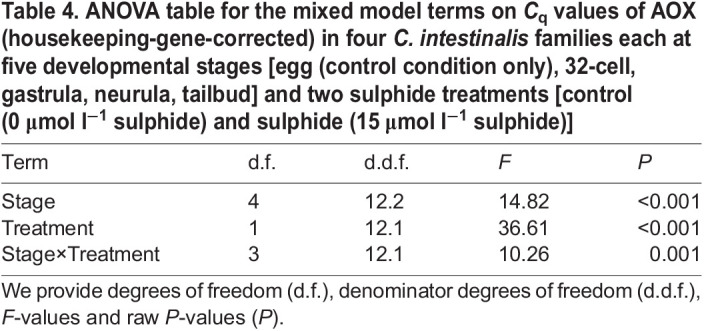
ANOVA table for the mixed model terms on *C*_q_ values of AOX (housekeeping-gene-corrected) in four *C. intestinalis* families each at five developmental stages [egg (control condition only), 32-cell, gastrula, neurula, tailbud] and two sulphide treatments [control (0 μmol l^−1^ sulphide) and sulphide (15 μmol l^−1^ sulphide)]

**Table 5.**
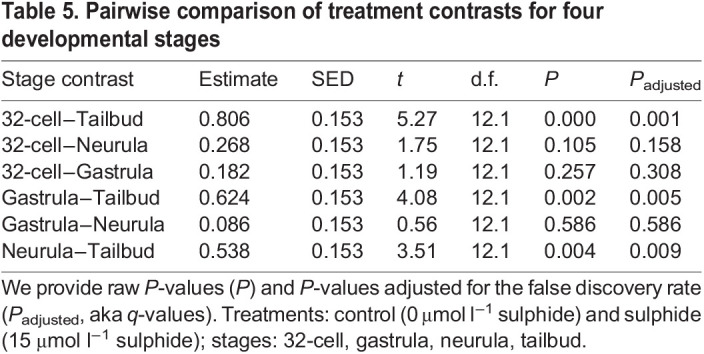
Pairwise comparison of treatment contrasts for four developmental stages

Because of the changing housekeeping gene mRNA levels across developmental stages, the underlying cause of this increase cannot be fully pinpointed (either an increase in *AOX* transcript levels or a decrease in the average housekeeping gene transcript levels with development).

## DISCUSSION

In this study, we investigated whether AOX is crucial for the development and survival of the highly invasive marine tunicate *C. intestinalis* under sulphide stress. To do so, we combined an MO-based knock-down approach and studied the transcriptional response of AOX. First, we demonstrated that early developmental success decreases with increasing sulphide concentrations. Second, we provided evidence that AOX is required for sulphide tolerance. Finally, we showed that sulphide elicits an increased relative amount of *AOX* transcripts. Together, these results provide support for the idea that the presence of AOX in *C. intestinalis* increases its sulphide tolerance during development and may thereby contribute to its invasive potential.

The determined LC_50_ of 15.2 μmol l^−1^ sulphide and the fully stalled development at a concentration of 50 μmol l^−1^ (Fig. 1) are compatible with known COX-inhibiting sulphide concentrations. Specifically, while the isolated COX enzyme is half-maximally inhibited at ∼0.2 µmol l^−1^ H_2_S, intact cells display a concentration of ∼30 µmol l^−1^ sulphide at which COX is inhibited (Leschelle et al., 2005; Petersen, 1977; Yong and Searcy, 2001). Because *C. intestinalis* embryos closely resemble intact cells, our results are consistent with previously estimated limits, suggesting that AOX is recruited when water sulphide levels are above known COX-inhibiting concentrations. Interestingly, the tolerance of *C. intestinalis* to sulphide is two orders of magnitude higher than that of eggs, fry and juveniles of non-AOX hosting fish species such as walleye, northern pike, sucker and rainbow trout (Adelman and Smith, 1970; Smith and Oseid, 1972, 1974). While the maximum possible safe level of sulphide for fish eggs lies between 0.41 and 0.53 μmol l^−1^, it is between 0.11 and 0.18 μmol l^−1^ for yolk-sac fry. Our results also align with the suggestions that developmental stages of many marine invertebrates are more sensitive to stress compared with their adult stages (Pineda et al., 2012; Ringwood, 1992). To our present knowledge, adult *C. intestinalis* can withstand much higher sulphide concentrations of up to 300 μmol l^−1^ than determined here for embryos (Saari et al., 2019). The underlying reason may be the limited repertoire of stress responses in embryos, which exclusively rely on cellular response mechanisms, whereas adults may employ additional behavioural and structural coping mechanisms. This illustrates very well the importance of taking all life stages into account, both when studying the ability of a species to survive in response to environmental stressors and predicting environmental effects on species and ecological communities.

Understanding the underlying mechanisms for coping with environmental stressors, including elevated H_2_S, is the basis for predicting ecological communities in the future (Hofmann and Todgham, 2010). In this context, AOX plays an interesting role. AOX is suggested to have fulfilled an important role in the evolution of animals and shaping ecological communities. The appearance of AOX can be dated back to before the great oxygenation event approximately 2.4 billion years ago, a very stable sulphide-rich era (Weaver, 2019). During this time, AOX is suggested to have allowed for metabolic flexibility and hence the rise of mitochondria and the first eukaryotes, and ultimately the rise of the first metazoans after a second (Neoproterozoic) oxygenation event approximately 800 million years ago. After this event, oxygen levels rapidly increased and the environment changed, yielding stable oxygenated conditions (Olson and Straub, 2016; Weaver, 2019). Current sulphide-rich environments including sediments, water columns and areas around vents are found in marine and freshwater environments around the globe. For example, H_2_S concentrations can reach up to 15,000 μmol l^−1^ in the pore waters of sediments off the coast of California, up to 6500 μmol l^−1^ around the hot vents of the East Pacific rise, or up to 800 μmol l^−1^ in freshwater springs in Mexico (Bagarinao, 1992; Oeschger and Vetter, 1992; Rosales Lagarde et al., 2014). More temporary, but recurring, sulphide-rich events have been observed off the coast of Peru with up 6 μmol l^−1^ sulphide (Schunck et al., 2013) and Namibia (up to 30 μmol l^−1^ sulphide), which lead to regular mass mortalities of fish (Copenhagen, 1953; Lavik et al., 2009). Furthermore, it is expected that areas of toxic H_2_S concentrations will increase in abundance, size and severity in many marine environments owing to the increase in anoxic waters (Schobben et al., 2015), which can be expected to affect ecosystems via large animal kills. Interestingly, and as one of the worst-case scenarios, the end-Permian marine mass extinction has been linked to anoxic and sulphide-rich oceanic conditions (Schobben et al., 2015).

To our current and best knowledge, this is the first functional study of AOX in an animal species. This, however, also impedes a direct comparison of our results with those of other studies. Nonetheless, the importance of AOX in the sulphide response was suggested previously. A study on mitochondria of a polychaete, the lugworm (*Arenicola marina*), found a strong indication that the alternative pathway via AOX is involved in the oxidation of sulphide (Hildebrandt and Grieshaber, 2008). Specifically, under elevated sulphide levels, oxygen consumption was high while no ATP was produced, and this reaction was completely blocked when AOX was inhibited (Hildebrandt and Grieshaber, 2008). These latter results may also partially explain why *A. marina* can survive quite high sulphide concentrations of up to 10 mmol l^−1^ (Groenendaal, 1980). A study on another polychaete, the echiuran worm (*Urechis unicinctus*), found that *AOX* mRNA levels in the body wall and hindgut tissue increased with both sulphide concentration and sulphide exposure time (Huang et al., 2013). In a previous study on adult *C. intestinalis*, *AOX* mRNA levels were increased in heart and neural complex under elevated sulphide concentrations (100 and 300 μmol l^−1^; Saari et al., 2019). A few additional studies examined the role of AOX under other environmental conditions, such as copper and cadmium exposure, hypoxia, anoxia and extreme temperatures. Interestingly, copper and cadmium, both COX inhibitors, do not elicit a response in *AOX* mRNA in *C. intestinalis* (see appendix of Saari et al., 2019). However, under both hypoxic and anoxic conditions, *AOX* mRNA levels are increased in the Pacific oyster (*Crassostrea gigas*), the freshwater mussel (*Diplodon chilensis*) and also in *C. intestinalis* adults (Saari et al., 2019; Sussarellu et al., 2012; Yusseppone et al., 2018). Notably, in *C. intestinalis* adults, the combined effects of hypoxia and sulphide exposure appeared to be additive for *AOX* mRNA levels (Saari et al., 2019). These findings support the idea that AOX may have played an evolutionarily important role under the sulphide-rich and oxygen-poor conditions during the Proterozoic era and allowed for the appearance of metazoans (Weaver, 2019). Only recently has the effect of temperature on the protein level of AOX been investigated in the copepod *Tigriopus californicus* (Tward et al., 2019). That study detected increased AOX protein levels under cold and heat stress. Interestingly, both the Pacific oyster and the freshwater mussel are also considered invasive species, which supports our suggestion that AOX may indeed be an additional mechanism in slow or sessile marine organisms to cope with a variety of environmental factors and that it may give those species an advantage under future ocean conditions.

Among species lacking the *AOX* gene, sulphide tolerance varies enormously but can also be quite high (Bagarinao, 1992; Grieshaber and Völkel, 1998). Cellular mechanisms, other than AOX, that explain sulphide tolerance include the sulphide-quinone oxidoreductase (SQR)-related sulphide oxidation as present in most domains of life (Theissen et al., 2003), the H_2_S-resistant COX such as found in the shortfin molly (*Poecilia mexicana*), and the H_2_S-binding haemoglobins of the giant tube worm (*Riftia pachyptila*) and the California killifish (*Fundulus parvipinnis*) (Bagarinao and Vetter, 1992; Zal et al., 1998). Using H_2_S-binding haemoglobins, the California killifish, for example, has an 8 h LC_50_ of 300 μmol l^−1^ H_2_S (Bagarinao, 1992).

To better understand the control of the AOX response to sulphide, we also investigated the transcriptional response of *AOX* to sulphide exposure at different developmental stages. Unfortunately, changes of the two housekeeping gene mRNA levels across developmental stages prevented us from being able to make clearer statements on the change of *AOX* mRNA across developmental stages. This exemplifies the crucial need of a careful analysis of housekeeping gene responses, particularly during dynamic biological processes, such as development, to allow for more reliable conclusions on a target gene response. However, we were able to draw conclusions about the difference of *AOX* mRNA levels between control and sulphide conditions at each developmental stage. We found no difference in *AOX* mRNA levels between sulphide and control conditions at the 32-cell stage, whereas the *AOX* mRNA levels were increasingly higher under LC_50_ sulphide exposure compared with no sulphide exposure at the later stages (Fig. 3). Further, the sulphide effect was clearly higher for the last stage studied, the tailbud stage, compared with the three earlier stages. These results agree with our findings that AOX indeed plays a crucial role in *C. intestinalis* survival under sulphide exposure, and is very likely controlled at the transcriptional level.

In summary, we showed that the *AOX* gene is not required for embryonic development in normal environmental conditions. However, we demonstrated a clear link between sulphide tolerance and AOX, which appears to be regulated, at a minimum, at the level of gene transcription. On a larger scale, these results provide a valuable insight into how fluctuations in sulphide, which are expected to increase under climate change, may affect marine ecosystems. As sulphide exposure increases in frequency and severity, it becomes more and more important to understand whether and how species are affected by sulphide. Many of the AOX-hosting animal species are sessile or slow-moving marine invertebrates with free floating larval stages, making them particularly susceptible to changes in sulphide as they cannot actively avoid the stressor; however, these species comprise many successful invasive species. Our finding about the role of AOX in survival under sulphide stress in *C. intestinalis* embryos provides the opportunity to extrapolate to other AOX-hosting species, which we expect to have similar competitive advantages under sulphide stress. Lastly, we hypothesize that AOX is an important protein that mitigates not only sulphide but also other environmental stressors affecting aerobic energy production and under whose presence AOX-possessing species may be more competitive as a result.
